# *trans*-Bis(nitrato-κ*O*)tetra­kis­(1-vinyl-1*H*-imidazole-κ*N* ^3^)copper(II)

**DOI:** 10.1107/S1600536812030607

**Published:** 2012-07-10

**Authors:** Fatih Şen, Ramazan Şahin, Ömer Andaç, Murat Taş

**Affiliations:** a Kilis 7 Aralık University, Vocational High School of Health Services, Department of Opticianry, 79000 Kilis, Turkey; b Ondokuz Mayıs University, Arts and Sciences Faculty, Department of Chemistry, 55139 Samsun, Turkey; c Giresun University, Arts and Sciences Faculty, Department of Chemistry, 28000 Giresun, Turkey

## Abstract

In the title compound, [Cu(NO_3_)_2_(C_5_H_6_N_2_)_4_], the Cu^II^ ion is located on an inversion centre. It features a Jahn–Teller-distorted octa­hedral coordination geometry, defined by four N atoms of four 1-vinyl­imidazole ligands in the equatorial plane and two nitrate O atoms in the axial positions. The nitrate anion is disordered over two sets of sites in a 0.801 (6):0.199 (6) ratio. In the crystal, the complex mol­ecules are linked by weak inter­molecular C—H⋯O and C—H⋯π inter­actions.

## Related literature

For applications and characterisation of related compounds, see: Sundberg & Martin (1974 ▶); Kurimura *et al.* (1994 ▶); Baran (1999 ▶); Zhao (2008 ▶).
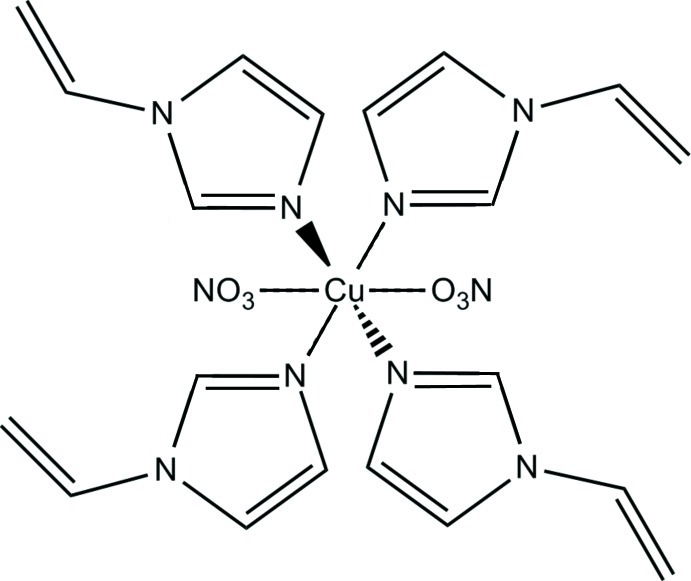


## Experimental

### 

#### Crystal data

[Cu(NO_3_)_2_(C_5_H_6_N_2_)_4_]
*M* *_r_* = 564.03
Monoclinic, 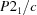

*a* = 8.9415 (4) Å
*b* = 8.7618 (3) Å
*c* = 16.3172 (6) Å
β = 102.281 (4)°
*V* = 1249.10 (8) Å^3^
*Z* = 2
Mo *K*α radiation
μ = 0.93 mm^−1^
*T* = 296 K
0.1 × 0.1 × 0.1 mm

#### Data collection

Oxford Diffraction SuperNova diffractometer
Absorption correction: multi-scan (*CrysAlis PRO*; Oxford Diffraction, 2007 ▶) *T*_min_ = 0.911, *T*_max_ = 0.911
6658 measured reflections
3820 independent reflections
2908 reflections with *I* > 2σ(*I*)
*R*_int_ = 0.018

#### Refinement

*R*[*F* ^2^ > 2σ(*F* ^2^)] = 0.041
*wR*(*F* ^2^) = 0.109
*S* = 1.03
3820 reflections
198 parameters
1 restraint
H-atom parameters constrained
Δρ_max_ = 0.26 e Å^−3^
Δρ_min_ = −0.32 e Å^−3^

### 

Data collection: *CrysAlis PRO* (Oxford Diffraction, 2007 ▶); cell refinement: *CrysAlis RED* (Oxford Diffraction, 2007 ▶); data reduction: *CrysAlis RED*; program(s) used to solve structure: *SHELXS97* (Sheldrick, 2008 ▶); program(s) used to refine structure: *SHELXL97* (Sheldrick, 2008 ▶); molecular graphics: *ORTEP-3 for Windows* (Farrugia, 1997 ▶); software used to prepare material for publication: *WinGX* (Farrugia, 1999 ▶) and *PLATON* (Spek, 2009 ▶).

## Supplementary Material

Crystal structure: contains datablock(s) global, I. DOI: 10.1107/S1600536812030607/wm2655sup1.cif

Structure factors: contains datablock(s) I. DOI: 10.1107/S1600536812030607/wm2655Isup2.hkl

Additional supplementary materials:  crystallographic information; 3D view; checkCIF report

## Figures and Tables

**Table 1:** Hydrogen-bond geometry (Å, °) *Cg*1 is the centroid of the imidazole (N1/C1/N2/C3/C2) ring.

*D*—H⋯*A*	*D*—H	H⋯*A*	*D*⋯*A*	*D*—H⋯*A*
C7—H7⋯O3*A*	0.93	2.41	3.273 (3)	155
C1—H1⋯O1*A* ^i^	0.93	2.52	3.273 (3)	139
C4—H4⋯O1*A* ^i^	0.93	2.38	3.217 (4)	149
C5—H5*B*⋯*Cg*1^ii^	0.93	2.78	3.6515	156
C10—H10*A*⋯*Cg*1^iii^	0.93	2.95	3.6691	136

Symmetry codes: (i) 
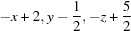
; (ii) 
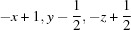
; (iii) 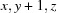.

**Fig. 1.**
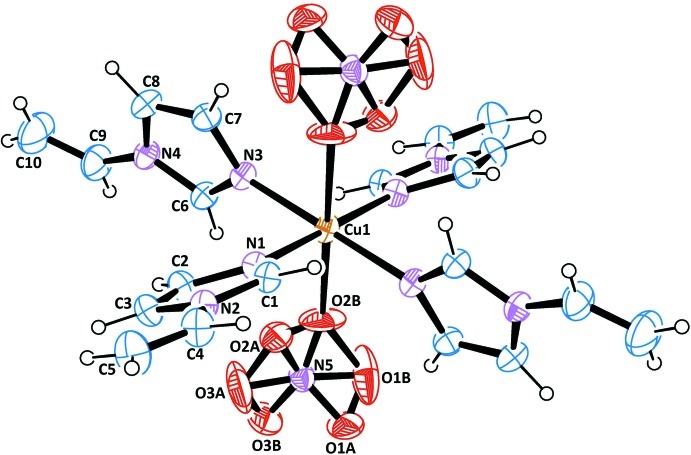
The molecular structure of (I), showing the atomic numbering scheme. Displacement ellipsoids are drawn at the 30% probability level. The disorder of the nitrate anion is shown, however only the shorter of the two Cu—O bonds is indicated.

**Fig. 2.**
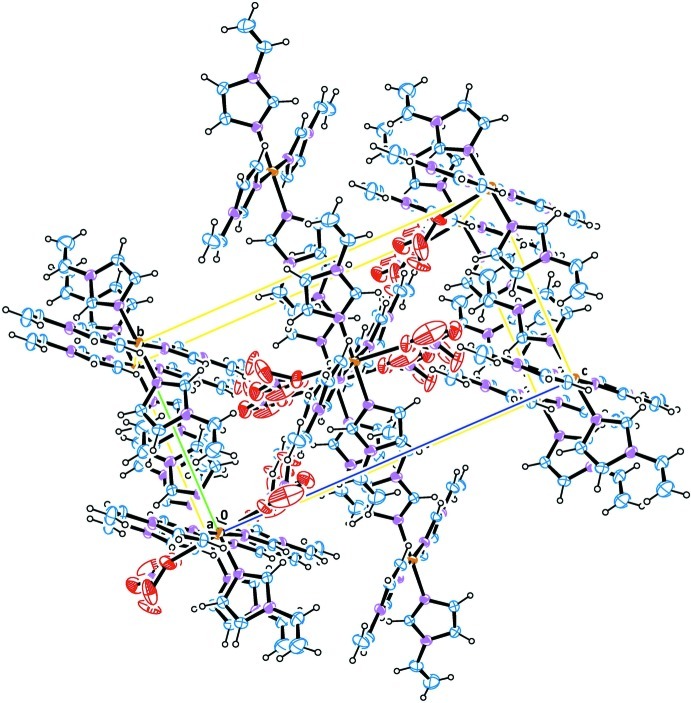
Packing of (I).

## supplementary crystallographic information

### Comment

Numerous complexes derived from *d*-block metals and imidazole ligands are well-known (Sundberg & Martin, 1974). A large number of investigations on the complexation of copper(II) with imidazole ligands and vinylimidazol ligands have been reported, with some of them reporting structures determined crystallographically (Kurimura *et al.*, 1994). We report here the crystal structure of the title compound, [Cu(C_5_H_6_N_2_)_4_(NO_3_)_2_], a mixed-ligand Cu^II^ complex, (I).

The molecular structure of compound (I) (Fig. 1) is similar to those of analogous derivatives (Baran, 1999). The Cu^II^ atom displays a Jahn-Teller distorted octahedral coordination geometry, with four N atoms from four 1-vinylimidazole ligands in the equatorial plane (with Cu—N1 and Cu—N3 bond lengths of 2.0091 (15) and 2.0147 (16) Å) and two nitrate molecules in axial positions (with Cu—O2B and Cu—O2A bond lengths of 2.531 (9) and 2.651 (3) Å). These bond lengths are comparable with those reported by Baran (1999).

The imidazole rings have a maximum deviation of 0.004 Å and 0.002 Å from planarity for atoms N2 and N3, respectively. The bond lengths and angles of the 1-vinyl-imidazole molecules in (I) show no significant differences to those of the related 2-chlorobenzoato structure (Zhao, 2008).

In the crystal structure (Fig. 2) the molecules are linked by two intermolecular C—H···O hydrogen bonds and two C—H···π hydrogen-bonding associations (Table 1).

### Experimental

Complex (I) was prepared from a mixtures of solutions containing copper(II) nitrate trihydrate (2.42 g, 10 mmol) and 1-vinylimidazole (1.98 g, 20 mmol) in 20 ml ethanol. The reaction mixture was stirred for 1 h, then 20 ml dissolved succinic acid (1.18 g, 10 mmol) was added. Then the reaction mixture solution was again stirred for 30 min and finally the solution was filtered. Blue single crystals of (I) were isolated after one day. Suitable crystals of (I) for structure determination were obtained from ethanol by slow evaporation (yield %55).

### Refinement

H atoms were positioned geometrically and treated using a riding model, fixing the bond lengths at 0.93 Å for the *sp*^2^ C atoms. The displacement parameters of the H atoms were constrained with *U*_iso_(H) = 1.2*U*_eq_(C). The nitrate anion was shown to be disordered over two sets of sites in a 0.801 (6):0.199 (6) ratio.

### Crystal data

**Table:**

[Cu(NO_3_)_2_(C_5_H_6_N_2_)_4_]	*F*(000) = 582
*M**_r_* = 564.03	*D*_x_ = 1.500 Mg m^−^^3^
Monoclinic, *P*2_1_/*c*	Mo *K*α radiation, λ = 0.71073 Å
Hall symbol: -P 2ybc	Cell parameters from 452 reflections
*a* = 8.9415 (4) Å	θ = 3.3–30.4°
*b* = 8.7618 (3) Å	µ = 0.93 mm^−^^1^
*c* = 16.3172 (6) Å	*T* = 296 K
β = 102.281 (4)°	Prism, blue
*V* = 1249.10 (8) Å^3^	0.1 × 0.1 × 0.1 mm
*Z* = 2	

### Data collection

**Table:**

Oxford Diffraction SuperNova diffractometer	3820 independent reflections
Radiation source: SuperNova (Mo) X-ray Source	2908 reflections with *I* > 2σ(*I*)
Mirror monochromator	*R*_int_ = 0.018
Detector resolution: 16.0454 pixels mm^-1^	θ_max_ = 30.5°, θ_min_ = 3.3°
ω scans	*h* = −12→12
Absorption correction: multi-scan (*CrysAlis PRO*; Oxford Diffraction, 2007)	*k* = −7→12
*T*_min_ = 0.911, *T*_max_ = 0.911	*l* = −22→23
6658 measured reflections	

### Refinement

**Table:**

Refinement on *F*^2^	Secondary atom site location: difference Fourier map
Least-squares matrix: full	Hydrogen site location: inferred from neighbouring sites
*R*[*F*^2^ > 2σ(*F*^2^)] = 0.041	H-atom parameters constrained
*wR*(*F*^2^) = 0.109	*w* = 1/[σ^2^(*F*_o_^2^) + (0.0433*P*)^2^ + 0.3004*P*] where *P* = (*F*_o_^2^ + 2*F*_c_^2^)/3
*S* = 1.03	(Δ/σ)_max_ = 0.001
3820 reflections	Δρ_max_ = 0.26 e Å^−^^3^
198 parameters	Δρ_min_ = −0.32 e Å^−^^3^
1 restraint	Extinction correction: *SHELXL97* (Sheldrick, 2008), Fc^*^=kFc[1+0.001xFc^2^λ^3^/sin(2θ)]^-1/4^
Primary atom site location: structure-invariant direct methods	Extinction coefficient: 0.0069 (15)

### Special details

**Table:**

Geometry. All e.s.d.'s (except the e.s.d. in the dihedral angle between two l.s. planes) are estimated using the full covariance matrix. The cell e.s.d.'s are taken into account individually in the estimation of e.s.d.'s in distances, angles and torsion angles; correlations between e.s.d.'s in cell parameters are only used when they are defined by crystal symmetry. An approximate (isotropic) treatment of cell e.s.d.'s is used for estimating e.s.d.'s involving l.s. planes.
Refinement. Refinement of *F*^2^ against ALL reflections. The weighted *R*-factor *wR* and goodness of fit *S* are based on *F*^2^, conventional *R*-factors *R* are based on *F*, with *F* set to zero for negative *F*^2^. The threshold expression of *F*^2^ > σ(*F*^2^) is used only for calculating *R*-factors(gt) *etc*. and is not relevant to the choice of reflections for refinement. *R*-factors based on *F*^2^ are statistically about twice as large as those based on *F*, and *R*- factors based on ALL data will be even larger.

### Fractional atomic coordinates and isotropic or equivalent isotropic displacement parameters (Å^2^)

**Table:**

	*x*	*y*	*z*	*U*_iso_*/*U*_eq_	Occ. (<1)
Cu1	1.0000	0.0000	1.0000	0.03972 (13)	
O1A	0.8327 (4)	0.0665 (4)	1.25474 (18)	0.0831 (11)	0.801 (6)
O1B	0.7861 (14)	0.031 (2)	1.1897 (19)	0.185 (15)	0.199 (6)
O2A	0.8731 (3)	0.0287 (3)	1.13193 (14)	0.0624 (8)	0.801 (6)
O2B	0.9871 (18)	0.0315 (12)	1.1525 (6)	0.092 (5)	0.199 (6)
O3B	0.9566 (15)	0.1935 (15)	1.2426 (8)	0.096 (5)	0.199 (6)
O3A	1.0438 (4)	0.1280 (6)	1.22800 (15)	0.116 (2)	0.801 (6)
N1	1.20913 (18)	−0.05380 (19)	1.06665 (9)	0.0390 (3)	
N2	1.39690 (17)	−0.1596 (2)	1.15773 (9)	0.0411 (4)	
N3	1.07291 (18)	0.21679 (18)	0.99548 (9)	0.0396 (3)	
N4	1.1880 (2)	0.42052 (19)	0.96108 (10)	0.0460 (4)	
N5	0.91506 (19)	0.0750 (2)	1.20390 (10)	0.0474 (4)	
C1	1.2438 (2)	−0.1520 (2)	1.12881 (11)	0.0410 (4)	
H1	1.1725	−0.2085	1.1500	0.049*	
C2	1.3466 (2)	0.0048 (2)	1.05604 (13)	0.0464 (5)	
H2	1.3573	0.0776	1.0162	0.056*	
C3	1.4628 (2)	−0.0580 (3)	1.11142 (12)	0.0467 (5)	
H3	1.5666	−0.0373	1.1173	0.056*	
C4	1.4720 (3)	−0.2583 (3)	1.22233 (13)	0.0547 (5)	
H4	1.4121	−0.3254	1.2457	0.066*	
C5	1.6189 (3)	−0.2611 (3)	1.25094 (16)	0.0709 (7)	
H5A	1.6820	−0.1954	1.2289	0.085*	
H5B	1.6612	−0.3288	1.2935	0.085*	
C6	1.1213 (2)	0.2877 (2)	0.93488 (11)	0.0430 (4)	
H6	1.1103	0.2500	0.8806	0.052*	
C7	1.1104 (2)	0.3105 (2)	1.06447 (11)	0.0459 (4)	
H7	1.0898	0.2904	1.1169	0.055*	
C8	1.1817 (3)	0.4360 (3)	1.04412 (13)	0.0526 (5)	
H8	1.2192	0.5171	1.0793	0.063*	
C9	1.2511 (3)	0.5242 (3)	0.90989 (19)	0.0670 (7)	
H9	1.2270	0.5083	0.8522	0.080*	
C10	1.3381 (4)	0.6372 (4)	0.9373 (2)	0.1013 (11)	
H10A	1.3647	0.6568	0.9945	0.122*	
H10B	1.3747	0.6997	0.8999	0.122*	

### Atomic displacement parameters (Å^2^)

**Table:**

	*U*^11^	*U*^22^	*U*^33^	*U*^12^	*U*^13^	*U*^23^
Cu1	0.04072 (19)	0.03473 (19)	0.03982 (18)	−0.00283 (14)	−0.00020 (12)	0.00636 (13)
O1A	0.081 (2)	0.104 (2)	0.0780 (17)	−0.0103 (18)	0.0492 (15)	−0.0192 (16)
O1B	0.047 (7)	0.116 (13)	0.40 (5)	−0.024 (8)	0.072 (15)	−0.016 (19)
O2A	0.0630 (17)	0.0759 (16)	0.0460 (11)	−0.0037 (13)	0.0064 (10)	−0.0199 (10)
O2B	0.144 (15)	0.076 (7)	0.076 (8)	0.012 (8)	0.069 (9)	−0.023 (6)
O3B	0.060 (7)	0.116 (9)	0.105 (8)	0.002 (6)	0.002 (6)	−0.079 (7)
O3A	0.074 (2)	0.213 (5)	0.0536 (14)	−0.074 (3)	−0.0024 (13)	0.012 (2)
N1	0.0414 (8)	0.0401 (8)	0.0343 (7)	−0.0005 (7)	0.0051 (6)	0.0040 (6)
N2	0.0405 (8)	0.0454 (9)	0.0348 (7)	0.0000 (7)	0.0025 (6)	0.0032 (7)
N3	0.0436 (8)	0.0355 (8)	0.0369 (7)	−0.0018 (7)	0.0025 (6)	0.0019 (6)
N4	0.0513 (9)	0.0370 (8)	0.0487 (9)	−0.0059 (8)	0.0087 (7)	0.0027 (7)
N5	0.0426 (9)	0.0559 (11)	0.0433 (8)	−0.0038 (8)	0.0085 (7)	−0.0015 (8)
C1	0.0403 (9)	0.0416 (10)	0.0390 (8)	−0.0032 (8)	0.0039 (7)	0.0042 (8)
C2	0.0466 (10)	0.0526 (12)	0.0417 (9)	−0.0010 (9)	0.0132 (8)	0.0090 (8)
C3	0.0399 (9)	0.0564 (12)	0.0452 (10)	−0.0006 (10)	0.0121 (8)	0.0061 (9)
C4	0.0536 (12)	0.0599 (14)	0.0458 (10)	−0.0003 (11)	0.0000 (8)	0.0153 (10)
C5	0.0552 (13)	0.0848 (19)	0.0652 (14)	0.0027 (14)	−0.0041 (11)	0.0248 (14)
C6	0.0500 (10)	0.0401 (10)	0.0374 (8)	−0.0025 (9)	0.0061 (7)	0.0007 (8)
C7	0.0516 (11)	0.0469 (11)	0.0385 (9)	0.0001 (9)	0.0075 (8)	−0.0008 (8)
C8	0.0643 (13)	0.0415 (11)	0.0498 (11)	−0.0055 (11)	0.0071 (9)	−0.0087 (9)
C9	0.0806 (18)	0.0566 (14)	0.0671 (15)	−0.0165 (13)	0.0230 (13)	0.0094 (12)
C10	0.135 (3)	0.0703 (19)	0.110 (2)	−0.041 (2)	0.051 (2)	−0.0040 (18)

### Geometric parameters (Å, º)

**Table:**

Cu1—N1^i^	2.0091 (15)	N2—C3	1.378 (3)
Cu1—N1	2.0091 (15)	N2—C4	1.418 (2)
Cu1—N3^i^	2.0147 (16)	N3—C6	1.316 (2)
Cu1—N3	2.0147 (16)	N3—C7	1.376 (2)
Cu1—O2B	2.531 (9)	N4—C6	1.336 (2)
Cu1—O2B^i^	2.531 (9)	N4—C8	1.375 (3)
Cu1—O2A^i^	2.651 (3)	N4—C9	1.428 (3)
Cu1—O2A	2.651 (3)	C1—H1	0.9300
O1A—O1B	1.10 (3)	C2—C3	1.342 (3)
O1A—N5	1.223 (3)	C2—H2	0.9300
O1A—O3B	1.612 (16)	C3—H3	0.9300
O1B—N5	1.191 (13)	C4—C5	1.297 (3)
O1B—O2A	1.34 (2)	C4—H4	0.9300
O2A—O2B	1.004 (15)	C5—H5A	0.9300
O2A—N5	1.223 (3)	C5—H5B	0.9300
O2B—N5	1.221 (10)	C6—H6	0.9300
O2B—O3A	1.490 (12)	C7—C8	1.347 (3)
O3B—O3A	1.036 (10)	C7—H7	0.9300
O3B—N5	1.231 (11)	C8—H8	0.9300
O3A—N5	1.226 (3)	C9—C10	1.280 (4)
N1—C1	1.316 (2)	C9—H9	0.9300
N1—C2	1.376 (3)	C10—H10A	0.9300
N2—C1	1.351 (2)	C10—H10B	0.9300
			
N1^i^—Cu1—N1	180.000 (1)	C6—N4—C9	124.9 (2)
N1^i^—Cu1—N3^i^	88.27 (6)	C8—N4—C9	128.2 (2)
N1—Cu1—N3^i^	91.73 (6)	C8—N4—Cu1	80.44 (12)
N1^i^—Cu1—N3	91.73 (6)	C9—N4—Cu1	151.17 (15)
N1—Cu1—N3	88.27 (6)	O1B—N5—O2B	113.3 (16)
N3^i^—Cu1—N3	180.000 (1)	O2A—N5—O1A	121.6 (3)
N1^i^—Cu1—O2B	106.0 (3)	O2A—N5—O3A	120.6 (2)
N1—Cu1—O2B	74.0 (3)	O1A—N5—O3A	117.7 (3)
N3^i^—Cu1—O2B	89.1 (3)	O1B—N5—O3B	123.1 (12)
N3—Cu1—O2B	90.9 (3)	O2B—N5—O3B	118.0 (9)
N1^i^—Cu1—O2B^i^	74.0 (3)	N1—C1—N2	110.89 (17)
N1—Cu1—O2B^i^	106.0 (3)	N2—C1—Cu1	142.94 (13)
N3^i^—Cu1—O2B^i^	90.9 (3)	N1—C1—H1	124.6
N3—Cu1—O2B^i^	89.1 (3)	N2—C1—H1	124.6
O2B—Cu1—O2B^i^	180.000 (3)	Cu1—C1—H1	92.5
N1^i^—Cu1—O2A^i^	95.32 (7)	C3—C2—N1	110.28 (17)
N1—Cu1—O2A^i^	84.68 (7)	C3—C2—Cu1	142.29 (14)
N3^i^—Cu1—O2A^i^	97.93 (7)	C3—C2—H2	124.9
N3—Cu1—O2A^i^	82.07 (7)	N1—C2—H2	124.9
O2B—Cu1—O2A^i^	157.8 (3)	Cu1—C2—H2	92.8
O2B^i^—Cu1—O2A^i^	22.2 (3)	C2—C3—N2	105.89 (17)
N1^i^—Cu1—O2A	84.68 (7)	N2—C3—Cu1	79.50 (10)
N1—Cu1—O2A	95.32 (7)	C2—C3—H3	127.1
N3^i^—Cu1—O2A	82.07 (7)	N2—C3—H3	127.1
N3—Cu1—O2A	97.93 (7)	Cu1—C3—H3	153.4
O2B—Cu1—O2A	22.2 (3)	C5—C4—N2	124.2 (2)
O2B^i^—Cu1—O2A	157.8 (3)	C5—C4—H4	117.9
O2A^i^—Cu1—O2A	180.000 (1)	N2—C4—H4	117.9
O1B—O1A—N5	61.4 (8)	C4—C5—H5A	120.0
O1B—O1A—O3B	101.9 (10)	C4—C5—H5B	120.0
N5—O1A—O3B	49.2 (4)	H5A—C5—H5B	120.0
O1A—O1B—N5	64.4 (11)	N3—C6—N4	111.49 (17)
O1A—O1B—O2A	121.6 (11)	N4—C6—Cu1	141.50 (13)
N5—O1B—O2A	57.3 (8)	N3—C6—H6	124.3
O1A—O1B—Cu1	128.6 (8)	N4—C6—H6	124.3
N5—O1B—Cu1	64.4 (12)	Cu1—C6—H6	93.8
N5—O2A—Cu1	135.6 (2)	C8—C7—N3	109.33 (17)
N5—O2B—Cu1	148.2 (9)	C8—C7—H7	125.3
N5—O3A—Cu1	71.46 (17)	N3—C7—H7	125.3
C1—N1—C2	105.69 (16)	C7—C8—N4	106.46 (18)
C1—N1—Cu1	127.61 (14)	N4—C8—Cu1	80.51 (12)
C2—N1—Cu1	126.69 (13)	C7—C8—H8	126.8
C1—N2—C3	107.24 (15)	N4—C8—H8	126.8
C1—N2—C4	125.05 (18)	Cu1—C8—H8	152.4
C3—N2—C4	127.69 (18)	C10—C9—N4	125.0 (3)
C3—N2—Cu1	81.48 (10)	C10—C9—H9	117.5
C4—N2—Cu1	150.75 (13)	N4—C9—H9	117.5
C6—N3—C7	105.73 (16)	C9—C10—H10A	120.0
C6—N3—Cu1	129.27 (13)	C9—C10—H10B	120.0
C7—N3—Cu1	123.86 (13)	H10A—C10—H10B	120.0
C6—N4—C8	106.98 (17)		

Symmetry code: (i) −*x*+2, −*y*, −*z*+2.

### Hydrogen-bond geometry (Å, º)

Cg1 is the centroid of the imidazole (N1/C1/N2/C3/C2) ring.

**Table:**

*D*—H···*A*	*D*—H	H···*A*	*D*···*A*	*D*—H···*A*
C7—H7···O3*A*	0.93	2.41	3.273 (3)	155
C1—H1···O1*A*^ii^	0.93	2.52	3.273 (3)	139
C4—H4···O1*A*^ii^	0.93	2.38	3.217 (4)	149
C5—H5*B*···*Cg*1^iii^	0.93	2.78	3.6515	156
C10—H10*A*···*Cg*1^iv^	0.93	2.95	3.6691	136

Symmetry codes: (ii) −*x*+2, *y*−1/2, −*z*+5/2; (iii) −*x*+1, *y*−1/2, −*z*+1/2; (iv) *x*, *y*+1, *z*.
